# The Role of T Cells in the Pathogenesis of Narcolepsy Type 1: A Narrative Review

**DOI:** 10.3390/ijms252211914

**Published:** 2024-11-06

**Authors:** Wenqi Xu, Wenting Ding, Yu Zhang, Shuanshuan Wang, Xianyu Yan, Yirui Xu, Xiaoying Zhi, Rongzeng Liu

**Affiliations:** Department of Immunology, College of Basic Medicine and Forensic Medicine, Henan University of Science and Technology, Luoyang 471003, China

**Keywords:** narcolepsy type 1, hypocretin, T lymphocytes, autoimmune diseases, molecular mimicry

## Abstract

Narcolepsy type 1 (NT1) is an uncommon, persistent sleep disorder distinguished by significant daytime sleepiness, episodes of cataplexy, and irregularities in rapid eye movement sleep. The etiology of NT1 is linked to the destruction of hypothalamic neurons responsible for the synthesis of the wake-promoting neuropeptide known as hypothalamic orexin. The pathophysiological mechanisms underlying NT1 remain inadequately elucidated; however, a model that incorporates the interplay of genetic predisposition, environmental influences, immune system factors, and a deficiency in hypocretin (HCRT) provides a framework for elucidating the pathogenesis of NT1. The prevalence of NT1 has been observed to rise following influenza A (H1N1) pdm09 and the administration of the Pandemrix influenza vaccine. The strong association between narcolepsy and the HLA-DQB1*06:02 allele strongly indicates an autoimmune etiology for this condition. Increasing evidence suggests that T cells play a critical role in this autoimmune-mediated HCRT neuronal loss. Studies have identified specific T cell subsets, including CD4^+^ and CD8^+^ T cells, that target HCRT neurons, contributing to their destruction. Clarifying the pathogenesis of NT1 driven by autoimmune T cells is crucial for the development of effective therapeutic interventions for this disorder. This review examines the risk factors associated with the pathogenesis of NT1, explores the role of T cells within the immune system in the progression of NT1, and evaluates immune-mediated animal models alongside prospective immunotherapeutic strategies.

## 1. Introduction

Narcolepsy type 1 (NT1) is a chronic sleep disorder characterized by an overwhelming tendency for excessive daytime sleepiness (EDS), episodes of cataplexy, and abnormalities in rapid eye movement sleep [1,2]. This condition may also be associated with phenomena such as sleep paralysis or sleep-shifting paralysis and hallucinations, frequent movements and awakenings during sleep, and weight gain [2,3,4]. The onset of NT1 typically occurs during adolescence [5], with an estimated incidence of about 1 in 100,000 persons-year [6]. Notably, 10–15% of individuals diagnosed with NT1 experience the onset of symptoms prior to the age of 10 [7]. Multiple studies indicate that NT1 is primarily attributed to the destruction of orexin-producing neurons rather than an inability to synthesize orexin itself [8,9,10]. Significant evidence supporting the notion of neuronal loss includes the observed reduction in hypothalamic levels of neuronal activity-regulated pentraxin and dynorphin, both of which are co-expressed in HCRT neurons [11]. Furthermore, additional research has demonstrated that NT1 results from the selective and irreversible loss of HCRT neurons within the hypothalamus in both human subjects and animal models [12,13,14,15].

Current epidemiological research indicates that both environmental and genetic factors play a role in the pathogenesis of NT1. A significant proportion of genetic predisposition for NT1 with cataplexy is associated with the *DQB1* locus. Individuals who are positive for the HLA-DQB1*06:02 allele exhibit a 251-fold increased risk of developing NT1 [16]. Following the extensive Pandemrix vaccination campaign conducted in 2009-2010, the incidence of NT1 increased by 5 to 14 times among children and adolescents and by 2 to 7 times in adults [17]. This finding suggests a significant association between the Pandemrix vaccination and an increased occurrence of NT1 [18]. In China, where the Pandemrix vaccine was not administered, the incidence of NT1 tripled following the H1N1 winter influenza pandemic of 2009-2010, indicating that an H1N1 infection itself may also elevate the susceptibility to NT1 [19]. Notably, all recent cases of NT1 that have arisen subsequent to H1N1 vaccination have been identified as carrying the HLA-DQB1*06:02 allele [16].

The robust genetic association with HLA-DQB1*06:02, alongside indications of immune dysregulation [20], and the increased incidence of disease following influenza vaccination implies that the loss of HCRT neurons may be attributable to both cellular and humoral immune responses. Such responses may become evident in individuals with a genetic predisposition when influenced by environmental triggers [21]. In the investigation of NT1 autoimmunity, several studies have reported significant alterations in autoantibodies among patients with NT1 when compared to a control group; however, none of these studies have conclusively demonstrated that these antibodies are specifically directed against NT1 [22,23,24,25]. Latorre et al. employed in vitro antigen stimulation alongside sensitive T cell library screening techniques to identify HCRT-specific CD4^+^ and CD8^+^ T cells present in the blood and cerebrospinal fluid (CSF) of affected individuals [21]. Furthermore, in vitro sensitization with H1N1 influenza virus antigens was observed to enhance the responses of CD4^+^ and CD8^+^ T cells to HCRT in children diagnosed with NT1, a response not seen in control children [26].

Overall, the prevailing evidence robustly supports the concept of T cell-mediated autoimmunity in patients with NT1, particularly highlighting the presence of autoreactive CD4^+^ and CD8^+^ T cells. A comprehensive understanding of the complex mechanisms by which T cells contribute to narcolepsy is essential for elucidating the immunopathogenesis of this multifaceted sleep disorder and may ultimately facilitate the development of targeted immunotherapeutic strategies. This review examines the immunological mechanisms underlying NT1 and the existing evidence that supports its classification as an autoimmune disorder. Additionally, it discusses relevant animal models and explores potential immunotherapeutic approaches.

## 2. Genetic Risk Factors for NT1

The HLA class II allele HLA-DQB1*06:02, in conjunction with *HLA*-DQA1*01:02, forms the DQ0602 heterodimer, a critical functional molecule for the presentation of antigens to T cells. This combination is nearly essential for the onset of NT1, although it is not solely sufficient by itself [27]. The association between NT1 and specific HLA alleles is pronounced (Table 1), as evidenced by the fact that 98% of patients with NT1 exhibiting cataplexy possess the DQB1*06:02 HLA allele, in contrast to only 25% of the general population [28]. Furthermore, both African-American and Caucasian-American individuals, regardless of cataplexy status, demonstrate a significantly elevated prevalence of DQB1*06:02 homozygotes compared to expected frequencies. The relative risk of developing NT1 is 2 to 4 times greater in DQB1*06:02 homozygotes than in heterozygotes across all patient cohorts [29].

In addition, several HLA alleles have been identified as contributing factors to the risk of developing NT1, including DQB1*03:01, DQA1*06, DRB1*04, DRB1*08, DPB1*05:01, DRB1*11, and DRB1*12 alleles. Conversely, numerous genome-wide association (GWA) studies have revealed that certain alleles, such as DQA1*01:03, DQB1*06:03, DPB1*04:01, DRB1*13:01, and DPB1*04:02, exhibit protective effects against the development of NT1 [30,31].

**Table 1:** The associations between HLA alleles and narcolepsy.

Gene	Effect Allele	Odds Ratio (95%CI)	*p*-Value	Study Population
*HLA-DQB1*	DQB1*03:01	2.48 (1.47–4.15)	<10^−3^	Japanese (2001) [30]
*HLA-DQB1*	DQB1*03:02	1.97 (1.57–2.47)	8.1 × 10^−9^	Japanese (2014) [32]
*HLA-DQB1*	DQB1*04:01	1.43 (1.15–1.79)	1.2 × 10^−3^	Japanese (2014) [32]
*HLA-DQB1*	DQB1*05:01	0.48 (0.31–0.77)	1.5 × 10^−3^	Japanese (2014) [32]
*HLA-DQB1*	DQB1*06:01	0.21 (0.09–0.52)	<10^−3^	Koreans (2007) [33]
*HLA-DQB1*	DQB1*06:02	51.18 (24.97–104.92)	<10^−4^	Czech (2016) [34]
*HLA-DQB1*	DQB1*06:03	0.17 (NA)	5.01 × 10^−12^	European (2014) [16]
*HLA-DQA1*	DQA1*01:02	4.98 (3.39–7.33)	<10^−3^	Japanese (2001) [30]
*HLA-DQA1*	DQA1*01:03	0.68 (0.10–0.56)	1.45 × 10^−4^	Chinese (2014) [31]
*HLA-DPB1*	DPB1*04:01	0.61 (0.47–0.80)	2.07 × 10^−4^	European and Chinese (2014) [31]
*HLA-DPB1*	DPB1*04:02	0.50 (0.35–0.70)	4.98 × 10^−5^	European (2014) [31]
*HLA-DPB1*	DPB1*05:01	1.76 (1.34–2.31)	4.71 × 10^−5^	Asian (2014) [31]
*HLA-DRA1*	DRA1*15:01	7.91 (NA)	3.13 × 10^−250^	Chinese (2014) [31]
*HLA-A*	A*11:01	1.49 (1.18–1.88)	7 × 10^−4^	European (2016) [35]
*HLA-B*	B*35:01	1.46 (1.13–1.89)	3.64 × 10^−3^	European (2016) [35]
*HLA-C*	C*04:01	1.34 (1.10–1.63)	3.23 × 10^−3^	European (2016) [35]

CI: confidence interval; NA: not available.

Subsequent research has identified a significant association between narcolepsy and polymorphisms in the T cell receptor alpha locus (*TRA*) [31,36]. The observed association between narcolepsy and the DQB1*06:02, along with *TRA* gene polymorphisms, indicates that the pathophysiology of the disorder may involve specific interactions among HLA, peptides, and *TRA* [37]. Additionally, polymorphisms in various other genes (Table 2), such as the purinergic receptor (*P2Y11*) [38], C-C motif chemokine receptors 1 and 3 (*CCR1-CCR3*) [39], cathepsin H (*CTSH*) [40], tumor necrosis factor superfamily member 4 (*TNFSF4*) [40], choline kinase beta (*CHKB*), and carnitine palmitoyltransferase 1B (*CPT1B*) [41,42], have also been associated with an increased susceptibility to narcolepsy. The interplay of immunogenetics and targeted damage to HCRT neurons suggests a potential autoimmune mechanism underlying the disease.

## 3. Environmental Risk Factors for NT1

A significant association has been identified between NT1 and the DQB1*06:02 allele, particularly in instances of cataplexy. However, the relatively low incidence of narcolepsy with cataplexy among carriers of this allele suggests the influence of additional environmental cofactors [47]. Furthermore, findings from twin studies indicate that concordance rates among identical twins are only 25 to 31 percent, underscoring the importance of environmental factors in the etiology of narcolepsy [48,49,50].

Between 2009 and 2010, subsequent to widespread vaccination campaigns targeting the H1N1 influenza virus, a notable rise in cases of juvenile-onset NT1 was observed in Finland and Sweden [51,52]. Further evidence indicated that within the first year following the administration of the Pandemrix vaccine, the incidence of NT1 was significantly elevated among children aged 4 to 19 years old. Moreover, a reduction in the levels of hypothalamic secretion in CSF was noted, with the lowest reported incidence being 10 cases per 100,000 individuals annually [53,54]. Furthermore, streptococcal infections are considered a substantial environmental factor contributing to the onset of NT1 [23]. However, in Chinese patients diagnosed with NT1, no evidence of Streptococcal infection was found, as indicated by the absence of elevated levels of antibodies against streptolysin O and DNase-B [55].

Various vaccines were deployed to combat the H1N1 influenza virus, with adjuvanted formulations being predominantly utilized in European nations [56]. An examination of the different vaccine brands indicated that only the AS03-adjuvanted Pandemrix vaccine (produced by GlaxoSmithKline, Brentford, UK) was identified as the sole vaccine associated with an increased risk of NT1, whereas the MF59-adjuvanted pandemic vaccines have not been linked to an elevated risk of NT1 [57]. The AS03 adjuvant is characterized as an oil-in-water emulsion adjuvant comprising α-tocopherol, squalene, and polysorbate 80 [58]. This adjuvant enhances the immune response by activating NF-κB [59], modulating the expression of certain chemokines and cytokines, and facilitating the migration of innate immune cells [60]. AS03 is capable of stimulating the specific immune response of CD4^+^ T cells, while endogenous CD8^+^ T cells in NT1 are responsible for the destruction of HCRT neurons, a process that necessitates the activation of CD4^+^ T cells to initiate the inflammatory response [27]. Nevertheless, the findings from multiple epidemiological studies suggest that the AS03 adjuvant alone is insufficient to induce the disease [57]. The attribution of NT1 solely to the AS03-adjuvanted vaccine remains a subject of debate. It is plausible that adjuvants may elicit generalized immune responses rather than specifically triggering autoimmunity related to narcolepsy [61]. Furthermore, the incidence of autoimmune disorders, aside from NT1, did not show a significant increase following the administration of the AS03-adjuvanted vaccine Pandemrix [62].

## 4. The Pathogenesis of NT1

Numerous researchers advocate for the autoimmune hypothesis in relation to NT1. Evidence supporting the immune-mediated mechanisms includes the detection of anti-Tribbles homolog 2 (TRIB2) [24] and anti-streptococcal antibodies [23] in patients with NT1, the seasonal patterns observed in the onset of NT1 [19], and the heightened incidence of NT1 following infections and vaccinations related to the H1N1 influenza virus [63]. The identification of elevated antibody levels against TRIB2 in individuals with NT1 marks a notable progression in the understanding of this condition, providing essential evidence of an inflammatory process in at least a subset of patients with NT1 [64]. Research conducted by H. Toyoda et al. has corroborated the increased prevalence and specificity of anti-TRIB2 autoantibodies among Japanese patients diagnosed with NT1. This finding implies that a particular subgroup within the NT1 spectrum may be influenced by an autoimmune mechanism mediated by anti-TRIB2 autoantibodies [65]. Furthermore, several studies have identified the presence of autoantibodies targeting HCRT receptors in patients with NT1. However, these investigations revealed no significant difference in the prevalence of these autoantibodies between patients with NT1 and control subjects [66], and the passive transfer experiments involving these autoantibodies did not successfully induce NT1 animal models [67], indicating a lack of association between narcolepsy and the autoantibodies associated with the HCRT neurotransmission system [66]. To date, no autoantibodies specific to HCRT neurons have been identified in patients with NT1 [68]. These results suggest that the autoimmune response associated with NT1 is predominantly driven by T cell-mediated cellular immunity, as opposed to being primarily mediated by antibodies.

**Figure 1:**
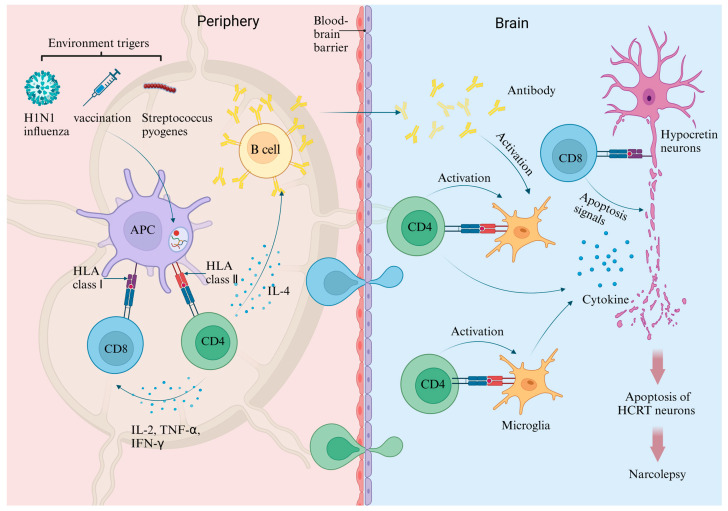
The autoimmune hypothesis of NT1. The key components in the pathogenesis of this disorder include T cells, microglia, and antibodies. Antigen-presenting cells (APCs) initially capture pathogens and present peptide fragments derived from these pathogens to naive T cells. Subsequently, B cells receive activation signals from these T cells, leading to their differentiation into plasma cells that produce antibodies. Activated T cells are capable of inducing inflammatory responses and secreting cytokines and chemokines, which can compromise the integrity of the blood-brain barrier (BBB), facilitating the infiltration of lymphocytes and antibodies into the central nervous system. Within the brain, HLA-II is exclusively expressed by microglia, whereas HLA-I is found in neurons. Cytotoxic CD8^+^ T cells have the ability to directly target and eliminate hypocretin-producing neurons, while both T cells and antibodies can activate microglial cells. The cytokines and chemokines released by microglia or T cells can further recruit additional immune cells, exacerbating damage to hypocretin neurons [70].

The critical role of HCRT deficiency in the pathogenesis of NT1 has led to the hypothesis the HCRT protein itself may serve as the autoantigen. Research has indicated that the amidated terminus of the secreted HCRT peptide (HCRT_NH2_) functions as an autoantigen in NT1 that specifically targets HCRT neurons [69]. Consequently, HCRT neurons may be the primary autoimmune targets for direct attacks and/or cytokine-mediated assaults by CD4^+^ or CD8^+^ T cells [70]. The proposed pathogenesis of NT1 suggests that the autoimmune destruction of HCRT neurons in the hypothalamus is a key factor, as illustrated in Figure 1. The infiltration of autoreactive T cells and antibodies into the central nervous system can trigger an immune response that results in the destruction of HCRT-producing neurons located in the lateral hypothalamus. This autoimmune response may be mediated by microglia or T cells that secret cytokines and chemokines, thereby recruiting additional immune cells and exacerbating damage to HCRT neurons or rendering them more susceptible to cytotoxic CD8^+^ T cells [70]. This hypothesis is further corroborated by genetic and environmental factors related to immune responses that are associated with the disease.

The onset of NT1 is associated with influenza A virus infection, with a particular fragment of the hemagglutinin (HA) protein from the Influenza A (H1N1) pdm09 virus identified as a potential trigger [71,72]. The HA epitope exhibits homology to HCRT_NH2_, and T cells that demonstrate cross-reactivity between these two epitopes are implicated in the autoimmune response, as evidenced by molecular simulation studies [69]. Furthermore, these antigens presented to specific HLA heterodimers are associated with narcolepsy-related genes and regulate the expression of specific T cell receptor fragments (*TRAJ24* and *TRBV4-2*) involved in the recognition of these antigenic T cell receptors, thereby suggesting a causal relationship [69].

## 5. Antigen-Specific T Cells in Human NT1

The targeted destruction of HCRT neurons within the hypothalamus strongly indicates an autoimmune response that specifically affects these cells [73]. Latorre et al. have demonstrated the presence of autoreactive memory CD4^+^ and CD8^+^ T cells in individuals diagnosed with narcolepsy using the T cell library method, which specifically targets autoantigens produced by HCRT-expressing neurons [21]. The autoreactive CD4^+^ T cells exhibited a polyclonal nature, targeting multiple epitopes. Notably, autoreactive clonotypes were consistently identified in the blood of the same patients and across different patients, while they were absent in healthy control subjects [21].

In patients with NT1 who received the H1N1 vaccine, there was a notable increase in the absolute counts of CD3^+^ and CD8^+^ T cells, as well as B cells, in comparison to patients with NT1 who were not vaccinated. These findings provide evidence for a global activation of T cells in individuals with NT1, suggesting a T cell-mediated autoimmune etiology for the disorder. However, the data do not indicate any detrimental effects associated with the H1N1 vaccination [74].

### 5.1. CD4^+^ T Cell

The strong association between NT1 and the DQB1*06:02 indicates a potential role of CD4^+^ T lymphocytes, thereby suggesting an autoimmune etiology for the disorder [75]. Furthermore, a comparative analysis reveals that patients with NT1 exhibit an elevated proportion of CD4^+^ memory T cells and regulatory T cells (Treg) in their peripheral blood relative to the general population, which signifies a heightened activation of the T cell system in these individuals [20,76]. The DQB1*06:02 gene encodes molecules that are synthesized and expressed on antigen-presenting cells, including macrophages, B cells, and dendritic cells, which are responsible for presenting antigenic peptides to CD4^+^ T cells [77]. It is important to note that CD4^+^ T cells do not possess cytotoxic capabilities, and HLA class II molecules are not present in neuron cells; thus, CD4^+^ T cells cannot directly mediate neuronal damage [78].

Research has demonstrated that the production of pro-inflammatory cytokines, specifically IL-2 and TNF-α, is markedly elevated in T cells of patients with NT1, suggesting an inflammatory component in the pathogenesis of the NT1 [73]. A sensitive T cell bank assay has revealed an increased prevalence of autoreactive CD4^+^ T cells in the bloodstream of individuals with narcolepsy [21]. When compared to a control group, patients with NT1 exhibited a greater propensity to secrete cytokines such as IL-2, tumor necrosis factor, IL-4, and IL-13, with these cytokine levels showing a positive association with both duration and patient age [73,79]. Furthermore, there was a significant increase in the frequency of circulating B cells and CD4^+^CXCR5^+^ follicular T cells among narcolepsy patients. Notably, the inducible T cell costimulatory molecule ICOS was selectively down-regulated on follicular T cells in these patients [80]. Additionally, autoreactive CD4^+^ T cells may contribute to localized inflammation and the disruption of the blood-brain barrier, thereby facilitating the activity of CD8^+^ T cells [81].

### 5.2. CD8^+^ T Cell

Neurons are known to express MHC class I molecules rather than MHC class II molecules, suggesting that the primary effector immune cells involved in their immune response may include MHC class I-restricted CD8^+^ T cells [35,82]. The signaling pathway mediated by IFN-γ enhances the expression of MHC class I on neurons, thereby facilitating their recognition by antigen-specific cytotoxic CD8^+^ T cells [83]. The autocrine or paracrine effects of IFN-γ further augment the mobility of cytotoxic lymphocytes in vivo, promoting the targeted killing of HCRT neurons. Evidence from transgenic mouse models expressing HA in HCRT neurons indicates that both antigen-specific Th1 cells and cytotoxic CD8^+^ T cells (CTLs) can induce hypothalamic inflammation. However, only CTLs are capable of inducing a selective loss of orexin-positive neurons, which parallels the pathology observed in human NT1. These findings support the hypothesis that CTL-mediated cytotoxicity, which is both direct and antigen-dependent, is a mechanism underlying the loss of orexin-positive neurons [84,85]. Notably, a patient with NT1 who succumbed to combined Ma2 antibody encephalitis exhibited significant infiltration of CD8^+^ T cells in the hypothalamus [84].

Research indicates that patients diagnosed with NT1 exhibit a heightened recognition of NT1-related proteins by CD8^+^ T cells when compared to healthy controls who are positive for DQB1*06:02. This enhanced recognition is characterized by both an increased frequency and a broader range of CD8^+^ T cell responses [86]. Comparable findings have been reported in various investigations concerning other autoimmune disorders. By employing barcoded HLA-peptide multimers, Pedersen et al. assessed the frequency of CD8^+^ T cells that are specific to seven potential neuronal autoantigens [86]. The results indicate a heightened autoreactivity of CD8^+^ T cells in individuals diagnosed with NT1 when contrasted with healthy control participants who are positive for DQB1*06:02. Furthermore, genome-wide association studies have identified an association with the *TRA,* which encodes the alpha chain of the TCR alpha/beta heterodimer [45]. The T cell receptor protein is known to interact with HLA class I molecules on cytotoxic CD8^+^ T cells as well as HLA class II molecules on helper CD4^+^ T cells [77].

In pathological conditions, the integrity of the blood-brain barrier is compromised, allowing autoreactive CD4^+^ T cells to infiltrate the central nervous system. This infiltration triggers an aberrant immune response that results in the destruction of lateral hypothalamic neurons. The presence of these CD4^+^ T cells or activated microglia leads to an elevation in the secretion of cytokines and chemokines. Additionally, CD8^+^ T cells may be activated to target HCRT neurons, which further initiates pro-apoptotic signaling pathways and contributes to the demise of these neurons [87].

### 5.3. T Cell Cross-Reactivity with Influenza Antigens (Molecular Mimicry)

The increase in cases of NT1 observed in 2009, following infection with the H1N1 virus and subsequent H1N1 vaccinations, indicates that mechanisms such as molecular simulation and bystander activation may play a significant role in the pathogenesis of the disease [88]. Molecular mimicry occurs when structural similarities between foreign and self-peptides promote the activation of autoreactive T cells by antigens derived from external sources in individuals who are genetically predisposed [89,90]. HA from the H1N1 influenza virus serves as the principal antigenic epitope, eliciting the host’s response to antigen during infection. Research has demonstrated that patients with NT1 exhibit elevated levels of HA antibodies in their bloodstream and that HA stimulation can enhance the T cell immune response to HCRT in these patients [71,72]. CD4^+^ T cells that recognize HCRT have been found to exhibit cross-reactivity with the HA protein from the H1N1 influenza virus [69].

A research team has established a murine model that facilitates the tracking and examination of T cell responses to the influenza virus HA, which is specifically expressed in orexinergic neurons. Vaccination with Pandemrix in this mouse model led to hypothalamic inflammation and the targeted destruction of orexin-producing neurons. Subsequent analyses of the roles of various T cell subsets indicated that HA-specific CD4^+^ T cells were essential for the onset of hypothalamic inflammation yet were inadequate in mediating the destruction of orexinergic neurons. In contrast, HA-specific CD8^+^ T cells did not initiate inflammation but acted as effectors in the destruction of orexinergic neurons. Further investigations have identified potential pathways that may be implicated in the disease process [14].

In a study examining the immune response in NT1, heightened reactivity was noted towards the influenza pHA_273–287_ (specific to pH1N1), as well as PR8 (H1N1 pre-2009 and H2N2)-specific NP_17–31_. Additionally, increased reactivity was observed towards the C-amidated version of HCRT_54–66_ and HCRT_86–97_ (HCRT_NH2_), while the native form did not elicit a similar response. Single-cell TCR sequencing indicated a shared CDR3β TRBV4-2-CASSQETQGRNYGYTF sequence in both HCRT_NH2_ and pHA_273–287_-tetramers stained TCRs, which suggests the occurrence of molecular mimicry [69]. Research conducted by Bernard-Valnet et al. demonstrates that an immunopathological process resembling NT1 can be triggered by immune cross-reactivity between a vaccine antigen and a neuronal self-antigen. This phenomenon is contingent upon the collaborative action of autoreactive CD4^+^ and CD8^+^ T cells in the progression of the disease. Such findings enhance our comprehension of the mechanisms and pathways that may contribute to the emergence of neurological side effects associated with vaccination, potentially with NT1 more broadly [14]. Furthermore, a 2013 investigation indicated that a specific fragment of the 2009 pH1N1 virus, which is also present in the vaccine, exhibits homology with HCRT_56–68_ and HCRT_87–99_, leading to molecular mimicry. The results revealed that patients with NT1 exhibited a higher frequency of autoimmune T cells targeting HCRT_56–68_, HCRT_87–99_, and pHA_275–287_ compared to the control group [91]. However, it is important to note that this article was subsequently retracted in the second year due to the inability to replicate the experimental results and the discovery of fabricated data [92].

Schinkelshoek et al. demonstrated that despite the structural homology observed between HLA-DQ6-hypocretin and H1N1 peptide complexes, T cell cross-reactivity was not identified. This finding suggests that the potential for cross-reactivity between H1N1-HA and HCRT peptides presented by the DQ0602 heterodimer is unlikely to play a role in the pathogenesis of NT1 [71]. Furthermore, it was noted that CD4^+^ T cell responses to H1N1 antigens significantly elevated in patients with NT1 when compared to control participants who were matched for HLA type and vaccination history [93].

## 6. Immune-Mediated Animal Models of NT1

Animal models play a crucial role in the investigation of the neuropathological mechanisms and potential therapeutic interventions for NT1. The development of immune-mediated NT1 animal models enables a more precise elucidation of the processes through which immune system dysfunction affects the HCRT system in individuals with NT1. This understanding is essential for identifying appropriate therapeutic targets aimed at the prevention and treatment of this condition.

Katzav et al. initially documented the administration of anti-TRIB2 antibody-positive mixed IgG into the lateral ventricle of the brain in immature mice. In comparison to the control group, which received IgG injections, there was a notable reduction in orexin-positive neurons; however, the experimental mice did not exhibit true cataplexy during the resting phase [94]. Furthermore, the observed loss of orexin neurons was not corroborated in subsequent investigations [25]. To date, there is a limited body of research regarding the role of humoral immunity in the induction of NT1, and the findings from these studies have not resulted in the manifestation of NT1 symptoms.

### 6.1. Orexin Gene Knockout Model

Narcolepsy is attributed to a deficiency in HCRT [95]. Research utilizing the orexin gene knockout mouse model has established that these mice maintain normal circadian rhythm regulation of sleep-wake behavior. Furthermore, the arousal-related monoamine neurotransmitters such as norepinephrine and histamine, which are significantly influenced by HCRT, were observed to be within normal ranges [96]. The findings from HCRT gene knockout mice indicate that HCRT neurons play a crucial role in the endogenous regulation of sleep and wakefulness. However, the compensatory upregulation of lateral hypothalamic melanocortin-concentrating hormone neurons can still modulate the sleep-wake circuitry, thereby complicating investigations into the expression of the orexin gene itself and its function in the promotion of sleepiness [97,98].

### 6.2. Lipopolysaccharide-Induced CCR3 Gene Knockout Model

Chemokine receptor 3 (*CCR3*) has been identified as a susceptibility gene linked to narcolepsy, with studies indicating a diminished expression level in the peripheral blood of individuals diagnosed with this condition [39]. As a G protein-coupled receptor, *CCR3* is instrumental in regulating the localization of immune cells and the chemokine gradients produced by inflammatory cells, thereby serving as an inflammatory mediator within the central nervous system. Research conducted by Toyoda et al. [99] revealed that *CCR3* knockout mice exhibited a reduced number of HCRT neurons compared to their wild-type counterparts; however, these knockout mice did not display any episode characteristic of narcolepsy. Subsequent administration of lipopolysaccharide (LPS) as an immune system stimulant to the *CCR3* knockout mice resulted in an extension of REM sleep duration, a reduction in non-rapid eye movement (NREM) duration, and an increase in seizure frequency. The LPS-induced dysfunction of *CCR3* in these mice provides a valuable model for investigating the immune-mediated degeneration of HCRT neurons triggered by environmental factors, thereby contributing to a deeper understanding of the immune pathogenesis associated with narcolepsy.

### 6.3. CD8^+^ T-Mediated HCRT Neuronal Injury Model

Bernard-Valnet et al. developed a mouse model expressing orexin-hemagglutinin (Orex-HA) mouse model that specifically targets the expression of the H1N1 influenza virus HA in HCRT neurons located in the hypothalamus [85]. The investigation into the contributions of various T cell subpopulations to this integrated disease mechanism was conducted through the transfer of individual T cell subpopulations and the antibody-mediated depletion of specific T cell subsets [14]. Prior research has indicated that while the adoptive transfer of autoreactive Th1 cells occurs within the hypothalamus, it does not facilitate the death of orexin neurons. Conversely, the adoptive transfer of cytotoxic CD8^+^ T cells has been shown to provoke inflammation and result in the death of orexin neurons. Although CD4^+^ T cells are capable of traversing the blood-brain barrier and instigating localized inflammation within the brain, they do not directly cause disease. The damage to HCRT neurons is primarily attributed to cytotoxic CD8^+^ T cells, which exhibit diminished cytotoxic efficacy following vaccination in the absence of assistance from CD4^+^ T cells.

HA-specific lymphocytes, including CD4^+^ T cells, CD8^+^ T cells, and B cells, were administered to murine models to enhance the immune response aimed at targeting and eliminating HCRT neurons that express HA [27,85]. The role of autoreactive CD4^+^ T cells, which are elicited by the influenza vaccine, is more nuanced. Specifically, CD4^+^ T cells are essential for facilitating the infiltration of autoreactive CD8^+^ T cells into the hypothalamus. In the absence of CD4^+^ T cells, although the metastatic CD8^+^ T cells exhibited significant proliferation, they were rendered ineffective, likely due to insufficient support from the vaccine-induced endogenous CD4^+^ T cells.

## 7. Immunotherapy

Currently, there is no established effective treatment for NT1, and the management of symptoms remains the primary strategy [100]. These approaches encompass a range of interventions, including intravenous immunoglobulin (IVIg), corticosteroids, plasma exchange, and immunoadsorption, among others. These therapies operate at various levels and do not specifically target distinct humoral or cellular immune pathways. Although they may exhibit some therapeutic benefits, they lack specificity for the disease itself. Furthermore, pharmacological agents aimed at T cell modulation have been evaluated in select patient populations.

### 7.1. Intravenous Immunoglobulin

Intravenous immunoglobulin (IVIg) therapy serves an immunomodulatory function by influencing both innate and adaptive immune responses, potentially exerting a beneficial effect on the autoimmune mechanisms implicated in the disease [101]. Research indicates that during the initial phase of NT1, clinical symptoms may experience temporary improvement following IVIg administration. However, the concentration of orexin in CSF remains unchanged [102]. Conversely, another investigation reported a complete normalization of HCRT levels in the CSF subsequent to IVIg treatment, implying that the initial impairment in HCRT release may be attributable to an inflammatory response rather than a depletion of HCRT-producing neurons [103,104]. If IVIg is administered at an early stage, it is possible that the release of HCRT could be progressively restored, with the normalization of HCRT levels serving as an indicator of the treatment’s potential efficacy.

In a separate investigation, individuals exhibiting negative CSF HCRT-1 levels between four months and one year post-onset were subjected to treatment. Notably, one of four patients demonstrated substantial improvement in EDS and cataplexy symptoms throughout the course of treatment, while the clinical profiles of the remaining patients did not exhibit consistent alterations [105]. Additionally, two other patients received IVIg therapy for a duration of five months shortly following the onset of NT1, which yielded only a transient enhancement in their condition [106].

### 7.2. Corticosteroids

Glucocorticoids are known to have immunomodulatory effects through various mechanisms and are commonly utilized in the management of inflammatory and autoimmune disorders [107]. Consequently, they are also considered in the treatment of NT1. Two male patients with a prolonged history of NT1 reported a cessation of EDS following the administration of prednisone. However, these observations were not substantiated by the measurements of CSF HCRT-1 levels or by subsequent sleep studies conducted after treatment. Given the extended duration between the onset of symptoms and the initiation of treatment, it is plausible that the stimulatory effects of corticosteroids may significantly influence the management of EDS [108]. Additionally, a case involving an 8-year-old boy who received prednisone two months post-acute onset of NT1 did not demonstrate any clinical improvement in sleep parameters [109]. This suggests that the damage to HCRT-producing neurons may be too extensive to reverse. Other reports have indicated the use of corticosteroids as adjunctive therapy for NT1. However, the outcomes of these interventions have generally been unfavorable [110].

### 7.3. Monoclonal Antibodies

Monoclonal antibodies utilized in the treatment of NT1 primarily consist of alemtuzumab and natalizumab. Alemtuzumab, specifically targeting the CD52 antigen present on T and B lymphocytes [111], can initiate the pro-apoptotic pathway in CD52-expressing cells through mechanisms such as antibody-dependent cellular cytotoxicity and complement-dependent cytotoxicity, leading to the depletion of circulating B and T lymphocytes. Consequently, this results in prolonged inhibition of CD4^+^ T cells [112]. Following treatment with alemtuzumab, only the symptoms of cataplexy were alleviated, while other symptoms showed no significant improvement.

Natalizumab, a recombinant monoclonal antibody, operates by inhibiting the α-4 integrin, a protein located on the surface of leukocytes [113]. Given that NT1 is likely attributable to pathogenic T cells, obstructing the migration of these T cells into the brain may mitigate the autoimmune assault on the HCRT neurons [114]. It is important to note that the onset of cataplexy symptoms indicates a loss of over 95% of HCRT neurons [115], indicating that the quantity of remaining HCRT neurons could be significantly diminished prior to the initiation of natalizumab treatment. Furthermore, not all patients with NT1 experience a rapid onset; some may develop cataplexy after prolonged periods of EDS, suggesting that certain individuals may possess a wider therapeutic window [116]. Additionally, natalizumab and analogous agents have the capacity to diminish the migration of T cells to the brain [114]. However, by the time treatment commences, pathogenic T cells are likely already present within the hypothalamus. The efficacy of natalizumab in preventing lymphocyte infiltration into the central nervous system underscores its potential as a therapeutic option for NT1.

## 8. Conclusions

Emerging evidence increasingly supports the autoimmune hypothesis for NT1, suggesting a role for the immune system in its pathogenesis. Despite this, immunomodulatory therapies like glucocorticoids, intravenous immunoglobulins, and plasma exchange, which primarily target the humoral immune system, have not shown consistent efficacy. The presence of autoreactive CD4^+^ and CD8^+^ T cells in patients indicates a potential role for T cells in the pathogenesis of the disease. However, the question of whether the inhibition of the autoimmune response can effectively halt disease progression is still a matter of debate. One therapeutic approach involves targeting autoantigen-HLA-II complexes on the surface of APCs using soluble TCR or TCR-like reagents, thus disrupting T cell-mediated autoimmunity with specificity. Another approach focuses on modulating T cell responses through monoclonal antibodies that interact with co-receptors, co-stimulatory molecules, or cytokines. Additionally, strategies like altering the TCR recognition of self-peptide/MHC complexes and applying modified peptide ligands to target pathogenic T cells offer alternative ways to disrupt disease progression. Future investigations should focus on evaluating the efficacy of T cell-targeted immunotherapy in animal models that replicate T cell-mediated destruction of hypothalamic neurons, as well as on conducting larger-scale clinical trials involving patients at risk for NT1. Elucidating the pathogenesis of NT1 not only aids in the development of effective therapeutic interventions for this disorder but also offers novel insights into the understanding of other diseases that are mediated by the autoreactive T cells.

## Figures and Tables

**Table 2:** The associations between non-HLA genes and narcolepsy.

Chr	Gene	rsID	Effect Allele	Odds Ratio (95%CI)	*p*-Value	Study Population
1	*TNFSF4*	rs7553711	C	1.33 (1.18–1.52)	4.08 × 10^−8^	European (2013) [40]
3	*CCR1/CCR3*	rs3181077	T	1.86 (1.40–2.47)	1.63 × 10^−5^	Japanese (2015) [39]
6	*EIF3G*	rs3826784	G	0.77 (0.68 0.88)	1.28 × 10^−4^	European (2015) [43]
7	*TRB*	rs2854536	G	0.78 (0.71–0.85)	3.87 × 10^−8^	Chinese and European (2013) [44]
10	*ZNF365*	rs10995245	A	1.23 (1.16–1.31)	1.24 × 10^−11^	Chinese and European (2013) [44]
14	*TRA*	rs1154155	C	1.87 (1.58–2.21)	1.90 × 10^−13^	North American, Asian and European (2009) [45]
15	*CTSH*	rs34593439	A	1.34 (1.21–1.46)	1.78 × 10^−8^	European (2013) [40]
19	*PPAN*	rs1551570	T	0.79 (0.69 0.90)	4.16 × 10^−4^	European (2015) [43]
19	*P2RY11*	rs2305795	A	1.33 (1.20–1.47)	5.19 × 10^−8^	European (2011) [38]
21	*IL10RB-IFNAR1*	rs2834188	A	0.78 (0.71–0.85)	3.87 × 10^−8^	Chinese and European (2013) [44]
22	*CPT1B*	rs5770917	C	1.56 (1.12–2.15)	3.6 × 10^−3^	Japanese (2009) [46]

Chr: chromosome, CI: confidence interval.
